# Mechanical modification and ultrasound-assisted osmotic dehydration for mass transfer intensification during blueberry convective drying

**DOI:** 10.1016/j.ultsonch.2026.107855

**Published:** 2026-04-15

**Authors:** Meliza Lindsay Rojas, Maria Namoc, Karla Ramirez, Alberto Claudio Miano

**Affiliations:** a Centro de Investigación Avanzada en Agroingeniería, Universidad Privada del Norte (UPN), the Republic of Peru; b Escuela de Ingeniería Agroindustrial, Universidad Privada del Norte, the Republic of Peru

**Keywords:** Blueberry valorization, Ultrasound, Drying pre-treatments, Viscoelasticity, Anthocyanins, Antioxidant capacity

## Abstract

This study investigated the use of mechanical modification (MM) and ultrasound-assisted osmotic dehydration (OD + US) as process-intensification strategies before convective drying of blueberries (*Vaccinium corymbosum* L.). MM (cutting and perforation) were combined with osmotic dehydration at 40–60°Brix, with ultrasound applied at 37 kHz and 33.86 W/L to enhance mass transfer. Peleg modeling revealed that ultrasound increased water loss and solute gain during OD, attributed to cavitation-induced microchannel formation and enhanced solvent penetration through the berry skin. Although MM facilitated initial mass transfer, ultrasound promoted additional structural disruption, particularly in cut samples, as evidenced by viscoelastic weakening and altered rehydration behavior. Drying kinetics, described by the Page model, showed that pre-treatments reduced the time required to reach 12% moisture by over 30%, indicating effective process intensification. Bioactive compounds decreased after drying; however, ultrasound-assisted treatments retained higher levels after OD compared to non-sonicated samples, suggesting reduced oxidative degradation during mass transfer. Sorption isotherms fitted to the GAB model enabled shelf-life prediction, demonstrating extended stability under aluminum laminate packaging. The combined perforation and OD + US treatment at 50°Brix provided the most favorable balance between drying efficiency and quality retention. Overall, results demonstrate that ultrasound acoustic cavitation combined with MM and OD enhances mass transfer, modifies microstructure, and improves dehydration performance in blueberries.

## Graphical abstract

**Figure:**
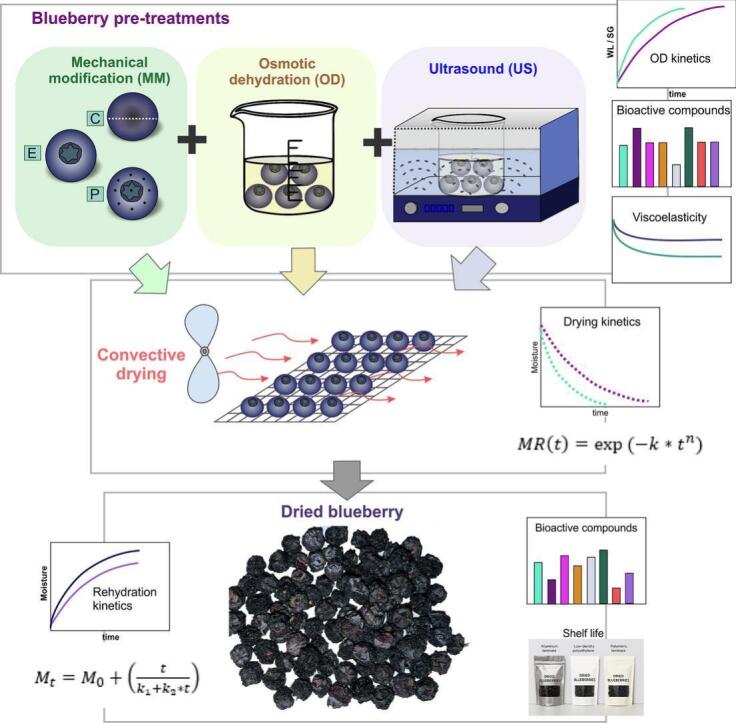

## 1 Introduction

Blueberry (*Vaccinium corymbosum L*.) is globally recognized for its attractive sensory profile and abundance of bioactive compounds [1]. These compounds have been associated with multiple health benefits, including antioxidant, anti-inflammatory, anticancer, and anti-aging effects [2], [3]. In particular, the high content of anthocyanins, flavonoids, and phenolic acids positions blueberries among the foods with the highest antioxidant capacity, associated with visual, neuronal, and cardiovascular health [4]. Consequently, blueberries are widely demanded by both the food and pharmaceutical industries.

Nevertheless, a significant proportion of harvested blueberries is lost throughout the supply chain, generating considerable discards or by-products during harvesting, processing, and exporting. During harvest, discards mainly occur when fruits do not meet increasingly strict market standards, particularly as Peruvian supply exceeds global demand [5]. During post-harvest operations of export blueberries, nearly 20% of the 146,000 tons produced annually in Peru are discarded due to mechanical damage, overripening, or defects in handling and packaging [6]. Although most of these fresh blueberry discards are sold in local markets, their limited shelf life significantly hinders effective commercialization and utilization. As a result, identifying processing and value-added strategies is especially important. One approach involves repurposing blueberry discards in the beverage industry; however, this process still results in 20% to 30% by-products (mainly pomace consisting of skins and seeds), indicating that the fruit is not yet being fully exploited [7].

Therefore, drying has been proposed as a strategy to fully valorize these discarded blueberries by extending their shelf-life, facilitating their distribution and storage, and enabling their incorporation into new food products. However, conventional hot-air drying presents challenges due to the presence of a hydrophobic waxy layer on the epidermis of many berries, which decreases water vapor permeability and restricts mass transfer [8]. In the specific case of blueberries (*Vaccinium corymbosum L*.), this cuticular barrier is particularly dense, which prolongs drying times and increases exposure to high temperatures, above 60°C, causing degradation of bioactive compounds and loss of quality (texture and color) [9]. Furthermore, the low water vapor permeability of this layer adversely affects dehydration kinetics [10] and raises energy consumption.

To overcome these limitations, various pre-treatments have been investigated, including chemical methods (hyperosmotic, alkaline, sulfite, and acidic solutions, as well as CO_2_, SO_2_, and O_3_ gases) and physical methods, either thermal (hot water, steam) or non-thermal (ultrasound, freezing, pulsed electric fields, high hydrostatic pressure) [11]. In blueberries (*Vaccinium corymbosum L*.), additional approaches such as osmotic dehydration (OD) [12], [13], [14], ultrasound [15], [16], [17], freezing [16], [17], pulsed electric fields [13], [14], CO_2_ [18], enzymatic treatments, ethyl oleate emulsions [8], [17], and mechanical modifications (laser or mechanical perforation) [17], [18], have been tested. Among these pre-treatments applied to blueberries, physical methods, OD, and ultrasound stand out as non-invasive, accessible, safe, and residue-free techniques, making them viable alternatives for scaling up equipment suitable for industrial applications. However, it is unknown how these pre-treatments, individually or in combination, affect key parameters such as drying kinetics, viscoelasticity, and the retention of bioactive compounds in discarded blueberries subjected to convective drying. In summary, it is not clearly established which type of pre-treatment offers the best balance between drying efficiency and functional preservation of the final product. This lack of evidence limits the ability to optimize dehydration processes that are efficient and respect the nutritional and sensory integrity of the fruit.

In this context, this research is based on the hypothesis that the application of mechanical modification (MM), OD, and ultrasound (US) as pre-treatments significantly improves the convective drying performance of blueberries and helps preserve their functional, structural, and storage properties. Under this premise, the study sets out to evaluate the effect of these pre-treatments on the drying time reduction and on key parameters such as viscoelasticity, retention of bioactive compounds, and stability of the final product.

## 2 MATERIALS AND METHODS

### 2.1 Raw material description

The proposal was developed using 15 kg of discarded blueberries of the Biloxi variety, obtained from a local market (Trujillo, Peru). It was verified that the product was free of foreign matter, had uniform coloration, and was free of bruises.

**Fig. 1:**
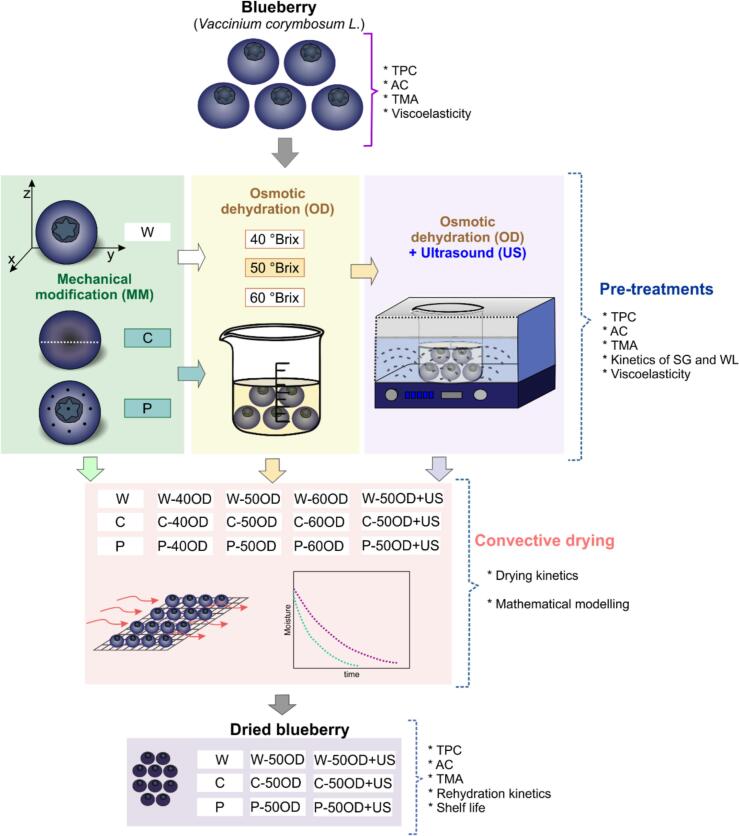
Representation of the experimental scheme carried out, which involved the analysis of fresh discarded blueberries, application of pre-treatments (MM, OD, US), drying, and analysis of the dried product. W: Whole, C: Cutting, P: Perforation, TPC: Total phenolic content, AC: Antioxidant capacity, TMA: Total monomeric anthocyanin, SG: Solid gain, WL: Water loss. Pre-treatment codes C and P indicate types of MM, pre-treatment codes #MM-#OD indicate type of MM followed by concentration of OD, finally codes #MM-50OD + US indicate type of MM followed by OD at 50°Brix assisted by ultrasound.

The application of three types of techniques as pre-treatments on discarded blueberries was evaluated (Fig. 1): mechanical modification (MM), Osmotic dehydration (OD), and ultrasound (US), whose application resulted in fourteen pre-treatments and one control (W, whole blueberry without pre-treatment).

### 2.2 Pre-treatment by mechanical modification (MM)

The blueberries were subjected to MM in their structure that served as pathways for mass transfer during the OD process and subsequent drying. Two types of MM treatments were considered:

Cutting (C): Partial longitudinal cuts were made in the blueberries using stainless steel knives, avoiding the berries from being split into halves.

Perforation (P): Perforations that go through the blueberry berries were made by using a stainless-steel cylinder with a diameter of 0.8 mm. A total of nine perforations were made in each blueberry, maintaining an equidistant distribution, using a prototype blueberry perforator designed by the researchers.

In addition, the whole (W) blueberries, without mechanical modifications, were subjected to subsequent pre-treatments and served as control samples.

### 2.3 Pre-treatment by osmotic dehydration (OD)

Discarded blueberries with or without MM were immersed in sucrose osmotic solutions at 3 concentrations (40°Brix, 50°Brix, and 60°Brix), these levels were selected because they are the functionally relevant range based on literature and preliminary screening tests (< 40°Brix, insufficient gradient; > 60°Brix, surface crust). The OD was performed for 6 h, which, in some cases, was extended up to 14 h to improve the mathematical fit of the Peleg model. A solid–liquid ratio of 1:10 (m/v), selected through previous tests to ensure that the mass transfer effects on the change in the concentration of the osmotic solution during the OD process are negligible. OD was described in terms of the two main mass transfer phenomena: solid gain (SG) and water loss (WL). Therefore, in blueberries, the percentage of SG and WL was evaluated throughout the OD processes, according to the procedure described by Polo Ruiz, Murga Mendoza, Obando Amaya, Lescano, Linares Luján, Sanchez-Gonzalez and Lindsay Rojas [19].

To calculate WL (${\mathrm{WL}}_{t}$ in g water/100 g sample) and SG (${\mathrm{SG}}_{t}$ in g solids/100 g sample) over the OD period, independent sample batches were sacrificed at each time. For each batch (∼20 g per replicate), the initial moisture (before immersion in the osmotic solution, $X_{w0})$, the initial weight of the blueberry samples (before immersion in the osmotic solution, $m_{0}$), the final weight (taken after a certain OD period, $m_{t}$), and the final moisture (taken after a certain OD period, $X_{\mathrm{wt}}$) were determined. At the end of each OD interval, the samples were washed to eliminate any remaining syrup from their surfaces and then dried with absorbent paper before measuring their weight and moisture content. This same procedure was applied after 6 h to all samples before proceeding to the convective drying stage.

Equations (1), (2) were used to calculate the WL and the SG, respectively [20]. Where, $X_{S0}$ and $X_{\mathrm{St}}$ correspond to their initial solid fraction and the solid fraction after a certain period (*t*) of OD, respectively.

(1) $${\mathrm{WL}}_{t}=\frac{\left(m_{0}.X_{w0}-m_{t}.X_{\mathrm{wt}}\right)}{m_{0}}*100$$

(2) $${\mathrm{SG}}_{t}=\frac{\left(m_{0}.X_{S0}-m_{t}.X_{\mathrm{St}}\right)}{m_{0}}*100$$

The Peleg model [21] (Equations (3), (4) was well applied to describe *WL* and *SG* during OD, as it provides not only a kinetic parameter but also a parameter related to mass transfer at equilibrium [20].

(3) $$\mathrm{WL}=\frac{t}{k_{1}^{w}+k_{2}^{w}∙t}$$

(4) $$\mathrm{SG}=\frac{t}{k_{1}^{s}+k_{2}^{s}∙t}$$

Where the reciprocals of $k_{1}^{w}$ (*WL^−1^∙ h*) and $k_{1}^{s}$ (*SG^-1^∙ h*) are related to the initial rates of *WL* and *SG*, respectively. Meanwhile, the reciprocals of $k_{2}^{w}$ (*WL^−1^*) and $k_{2}^{s}$ (*SG^-1^*) correspond to *WL* and *SG* at equilibrium, when *t*→∞.

An intermediate level of osmotic concentrations assessed during osmotic pretreatment was selected (50°Brix) for comparison with the application of ultrasound assisting osmotic pretreatment.

### 2.4 Ultrasound (US)-assisted OD

A high-power low-frequency ultrasound bath (33.86 W/L; 37 kHz) (Helmasonic, Germany) containing 15 L of water as the medium for acoustic energy transmission was used. Inside the ultrasound bath were placed the vessels containing the osmotic solution (50°Brix) and the blueberries with or without MM, in a solid–liquid ratio of 1:10 (m/v), the US-assisted OD was performed for 6 h. During the processing time, the temperature was kept at a controlled level between 26 ± 2°C. This was achieved by monitoring water temperature using temperature sensors. When the temperature approached the upper limit, a certain amount of water was extracted from the ultrasonic bath and replaced with cold water at 1°C in the same amount extracted.

The collection of weight and moisture data for the samples during US-assisted OD was conducted in accordance with the methodology outlined in section 2.3.

### 2.5 Convective drying

Convective drying was carried out at 50°C and 1.1 m/s using a forced convection oven (Memmert, Germany). Discarded blueberries were placed on a metal mesh to allow free movement of hot air over the entire surface of the samples. Initial moisture content (before drying) and final moisture content (after drying) were measured by completely drying the crushed samples at 105°C using a moisture analyzer (Ohaus, USA).

All moisture data on a dry basis (g water/g dry matter) during the drying period ($M_{t}$) were calculated by mass balance from sample weights recorded throughout the drying period and the final moisture. Based on the moisture data over time, the dimensionless moisture content (${\mathrm{MR}}_{t}$) (Equation (5) was calculated, where ($M_{t}$) is the dry basis moisture content during the drying process (*t*), ($M_{e}$) is the equilibrium moisture content (moisture after 60 h of drying time where no changes in mass were registered), and ($M_{0}$) is the initial moisture content of the sample before drying.

(5) $${\mathrm{MR}}_{t}=\frac{M_{t}-M_{e}}{M_{0}-M_{e}}$$

Drying curves were constructed based on the dimensionless moisture ratio ${\mathrm{MR}}_{t}$ versus the drying time (*t*). The Page model (Equation (6) was fitted to the drying data, as this is an empirical model commonly applied to describe drying processes [22], where k (h^-n^) and n (−) represent the drying rate and the dimensionless drying constant, respectively.

(6) $$MR\left(t\right)=exp\left(-k*t^{n}\right)$$

The Page model parameters were subsequently used to calculate the drying time required to reach a standardized final moisture content of 12% w.b. (0.136 g water/g dry matter) for all treatments, enabling consistent cross-treatment comparison.

### 2.6 Blueberry properties

#### 2.6.1 Viscoelastic properties

The viscoelastic properties were assessed using the stress-relaxation assay described by Rojas and Augusto [23]. In this procedure, the viscoelastic characteristics of berries were measured both before and after the application of pre-treatment. The assay involves applying a compression force (z-axis from Fig. 1) to the sample at a velocity of 0.2 mm/s until a displacement of 1 mm is reached. This distance is maintained for 30 s while the relaxation force is recorded to obtain a stress-relaxation curve. Testing was conducted with a Texture Analyzer TA.XT Plus (Stable Micro Systems Ltd., Surrey, United Kingdom) equipped with a 30 kg-f load cell and a 35 mm diameter aluminum cylinder probe (P/35R), with a smooth surface. The assay was performed at room temperature (22 ± 2°C).

Each berry was compressed perpendicularly to the calix-stem section. Strain (Ɛ) was determined by dividing the 1 mm displacement by the berry diameter. The diameter (y-axis from Fig. 1) of at least 20 berries was measured using a digital caliper (Mitutoyo, Japan). Stress was computed by dividing the relaxation force (kN) by the transversal area (xy plane from Fig. 1) of the blueberry, considering it as circular.

To describe the viscoelastic properties, the Generalized Maxwell Model [24], [25] was fitted to the experimental data of stress-relaxation. This model is composed of Maxwell elements (ME) arranged in parallel with an isolate elastic element. Each Maxwell element is composed by a Hookean spring (related to an elastic solid behavior) and a Newtonian dashpot (related to a viscous fluid behavior) arranged in series. In this work, it was considered two ME, this representation is presented in Equation (7), where $\sigma \left(t\right)$ is the stress as a function of time (*t*), *Ɛ* is the strain, $\xi_{i}$ represents the elastic module of each ME and $\tau_{i}$ represents the relaxation time of each ME.

(7) $$\sigma \left(t\right)=\varepsilon .\left(\xi_{e}+\xi_{1}.\exp\left(-\frac{t}{\tau_{1}}\right)+\xi_{2}.\exp\left(-\frac{t}{\tau_{2}}\right)\right)$$

Equation (8) was used to determine the viscosity value ($\eta_{i}$) of each ME.

(8) $$\eta_{i}=\tau_{i}.\xi_{i}$$

### 2.7 Bioactive compounds

#### 2.7.1 Obtaining sample extracts

Ethanolic extracts were prepared from raw, pre-treated, and dried blueberry samples to analyze total phenolic content (TPC), antioxidant capacity (AC), and total monomeric anthocyanins (TMA). For each extract, approximately 10 g of raw (82.5% moisture) or pre-treated samples (77.7% − 80.7% moisture), or 2 g of dried samples (10.5% – 25% moisture), was combined with 40 mL of 96% ethanol (J.T. BAKER), previously cooled at 1°C. The mixture was stirred for 1 h and subsequently vacuum-filtered using quantitative cellulose filter paper (Whatman No. 42). Extracts were collected in airtight, light-protected containers and stored under refrigeration at 1°C until further analysis.

#### 2.7.2 Total phenolic content (TPC)

For the determination of TPC, the Folin-Ciocalteu reduction method was employed. The TPC analysis was carried out based on the procedures reported by Colucci, Fissore, Rossello and Carcel [26], Moreno, Brines, Mulet, Rosselló and Cárcel [27], Rojas, Augusto and Cárcel [28], with some modifications. For the reaction, the extract (with dilutions and quantities adjusted as needed) was mixed with 100 µL of 2 M Folin-Ciocalteu reagent (MERCK) using a vortex mixer. After 4 min, 200 µL of 20% w/v sodium carbonate (MERCK) were added, and the mixture was kept for 60 min in darkness at room temperature. Subsequently, absorbance was measured at 765 nm (ThermoScientific, USA), distilled water was used to zero the spectrophotometer. The calibration curve was prepared using different amounts (from 10 to 90 µL) of 250 µg/mL gallic acid (GA) (3,4,5-trihydroxybenzoic acid) (MERCK) solution. The total phenolic content was expressed as GA equivalents (µg GAE/mg dry matter).

#### 2.7.3 Antioxidant capacity (AC)

The ABTS method described by Vieira, Borges, Copetti, Amboni, Denardi and Fett [29], with some modifications, was used and expressed as antioxidant capacity. The ABTS•+ radical was generated according to Re, Pellegrini, Proteggente, Pannala, Yang and Rice-Evans [30] by oxidation of ABTS (2,2′-azino-bis [3-ethylbenzothiazoline-6-sulfonic acid] ammonium salt, 7 mM) (MERCK) with potassium persulfate (2.45 mM) (MERCK). Ethanol (J.T. BAKER) 96% was used as the solvent for the ABTS•+ radical. Subsequently, the ABTS solution was prepared by adjusting its absorbance to approximately 0.700 a.u. at 734 nm using a UV–Vis spectrophotometer (ThermoScientific, USA). For the reaction, the ABTS solution, the extract (with dilutions and quantities adjusted as needed), and 96% ethanol were used. After the reaction, the mixture was left to stand for 20 min in the dark at room temperature, and then the absorbance was measured at 734 nm, ethanol 96% was used to zero the spectrophotometer. The calibration curve was constructed with different amounts of Trolox (6-hydroxy-2,5,7,8-tetramethylchroman-2-carboxylic acid, 0.5 mM; from 10 to 80 µL) (MERCK). The antioxidant capacity was expressed in mg of Trolox/g of dry matter.

#### 2.7.4 Total monomeric anthocyanins (TMA)

The total monomeric anthocyanin content in blueberries was determined according to the method proposed by Giusti and Wrolstad [31], Lee, Durst and Wrolstad [32]. The filtered sample was diluted with 0.025 M phosphate buffer solutions (pH = 1) and 0.4 M acetate buffer (pH = 4.5). The appropriate dilution factor was initially determined by diluting the sample with the pH 1 buffer solution so that the absorbance at 520 nm was within the range of 0.2 to 1.4 a.u.; a maximum dilution ratio of 1 part sample to 4 parts buffer solution was used to avoid exceeding the buffer capacity. Once the dilution factor was set, one part of the sample was diluted with phosphate buffer (pH = 1.0) and another part with acetate buffer (pH = 4.5), maintaining the same dilution factor. Subsequently, the absorbance of the solutions was measured at 518 nm and 700 nm using a spectrophotometer (ThermoScientific, USA), distilled water was used to zero the spectrophotometer. The concentration of TMA (Equation (9) was expressed as mg cyanidin-3-glucoside (cyd-3-glu) equivalents per g of dry matter (d.m).

(9) $$TMA\left(\frac{\mathrm{mg}}{\mathrm{gd}.m}\right)=\frac{A∙MW∙DF∙10^{3}}{\varepsilon ∙l}$$

Where, *A* = (A_520nm_ – A_700nm_) _pH 1.0_ – (A_520nm_ – A_700nm_) _pH 4.5_; *MW* (molecular weight) = 449.2 g/mol for cyanidin-3-glucoside (cyd-3-glu); *DF* = dilution factor; ∙l = optical path length of the cell (1 cm); *ε* = molar extinction coefficient (26 900 L∙mol^−1^∙cm^−1^, for cyd-3-glu); and 10^3^ = conversion factor for g to mg.

#### 2.7.5 Rehydration properties

Before rehydration, the dried blueberry samples were equilibrated to the same initial water activity (a_w_) to avoid its effect on rehydration kinetics [33]. This was achieved by storing the samples in an environment with 75% relative humidity, maintained using a saturated NaCl (CDH) solution. Rehydration was then performed at 30°C using tap water, with a solid-to-liquid ratio of 0.02  g/mL [34].

The process was performed as described in section 2.6.3. For the kinetics, the weight of the samples was recorded every 30 min throughout the rehydration process until approximately 10 h. The moisture content of each sample throughout the rehydration process was obtained by mass balance by considering the sample weight and its initial moisture content of the sample. The moisture content ($M_{t}$) in dry basis (d.b. = g water/g dry matter) during the process time (*t*) was plotted and the Peleg’s model [35] was fitted to the rehydration kinetics data (Equation (10).

(10) $$M_{t}=M_{0}+\left(\frac{t}{k_{1}+k_{2}*t}\right)$$

The Peleg’s model presented two parameters, $k_{1}$, related to the inverse of the rehydration rate [min·(g water/ g d.m.)^-1^] and $k_{2}$ (g water/ g d.m.)^-1^, related to the inverse of the maximum absorbed water.

#### 2.7.6 Shelf-life estimation of dried blueberries

The main factor determining the shelf life of a dehydrated product is its moisture content, which was selected as the key quality parameter in this study. The shelf-life of dried blueberries was estimated using Equation (11), which considers that the change in the product’s moisture content during storage is caused by the transfer of water vapor through the packaging material.

(11) $$\mathrm{SL}=\frac{\ln\left[Me-Mo\right)/\left(Me-Mc\right)]}{WVTR\left(\frac{A}{W}\right)\left(\frac{1}{I}\right)}$$

Where, Mo is the initial moisture content of the dried sample (g water/g dry matter), Me is the equilibrium moisture content under storage conditions (d.b., at 25°C and 75% relative humidity), Mc is the critical moisture content (d.b., corresponding to $a_{w}=0.5$, considered safe for storing dry products)[36], WVTR is the water vapor transmission rate through the packaging material (g/day·m^2^), A is the package surface area (m^2^), W is the mass of dry matter (g), and I is the slope of the isotherm between critical (Xc) and equilibrium moisture (Xe).

For packaging, 60 g of dried blueberries were considered per package. Three types of packaging materials were evaluated: low-density polyethylene (LDPE), polymeric laminate (PL) (PE/adhesive/EVOH/adhesive/PE), and aluminum laminate (AL), which showed WVTR values of 0.730, 0.201, and 0.072 g/day·m^2^, respectively [37].

To determine the critical and equilibrium moisture contents (Xc and Xe), adsorption isotherms were constructed at 25°C. Dried blueberry samples were placed in environments with different water activities (${\text{a}}_{w}$) ranging from 0.1 to 0.98, using saturated solutions of lithium chloride (MERCK), magnesium chloride (MERCK), lithium nitrate (MERCK), sodium bromide (MERCK), potassium iodide (MERCK), sodium chloride (CDH), and potassium chloride (MERCK), as well as sodium chloride solutions at 13.42% and 2.44% for ${\text{a}}_{w}$ values of 0.91 and 0.98, respectively. For ${\text{a}}_{w}$ levels above 0.6, thymol was used as a preservative. Samples were kept in these environments until equilibrium was reached for each ${\text{a}}_{w}$. Moisture content versus Me data were described using the GAB model (Equation (12), where Xe is the equilibrium moisture content, ${\text{a}}_{w}$ is water activity, $X_{m}$ is the monolayer moisture content (dry basis), and $C_{g}$ and K are constants of the GAB model.

(12) $$\mathrm{Xe}=\frac{X_{m}C_{g}Ka_{w}}{\left(1-Ka_{w}\right)\left(1-Ka_{w}+C_{g}Ka_{w}\right)}$$

### 2.8 Mathematical fitting and statistical analysis

The parameters of the mathematical models of Equations (3), (4) were obtained by nonlinear regression by using the Levenberg-Marquardt estimation method implemented in Statistica v.07 software (Statsoft, USA). In addition, the parameters of mathematical models of Equations (6), 7, 10 and 12 were obtained by iteratively fitting the models to the experimental data, minimizing the sum of squared errors (SSE in Equation (13) between the experimental and predicted values. A generalized reduced gradient algorithm, implemented in the 'Solver' tool of Excel software (Microsoft 365, USA), was used. Different initial parameter estimates were evaluated to detect possible convergence towards local optima.

(13) $$\mathrm{SSE}=\sum_{i=1}^{x}{\left((predicted)-(experiemental\right)}_{i}^{2}$$

All processes and analyses were carried out at least three times. For OD, drying kinetics and viscoelastic properties, factorial analysis of variance (ANOVA) followed by Tukey’s test was performed by considering MM (whole as control, cut and perforated) and OD (40°Brix, 50°Brix, 60°Brix) as factors. Further, students’ *t*-test was used to compare the means of pre-treatments without ultrasound (50OD) and with ultrasound (50OD + US). In addition, Principal Components Analysis (PCA) was performed to discriminate osmotic pretreatments according to their viscoelastic properties (Maxwell model parameters) and mass transfer characteristics during OD (WL and SG) and drying (Peleg model parameters). The analysis enabled to reduce response variables to only 2 factors or dimensions (DIM).

For rehydration properties and bioactive compounds (TPC, AC and TMA), ANOVA and Tukey’s test were performed considering MM (whole as control, cut and perforated) and OD (without, 50°Brix, 50°Brix + Ultrasound) as factors. All statistical analyses were conducted with a confidence level of 95% (α = 0.05) by using Statistica v.07 software (Statsoft, USA).

## 3 Results and discussion

### 3.1 Mass transfer description during pre-treatments

**Fig. 2:**
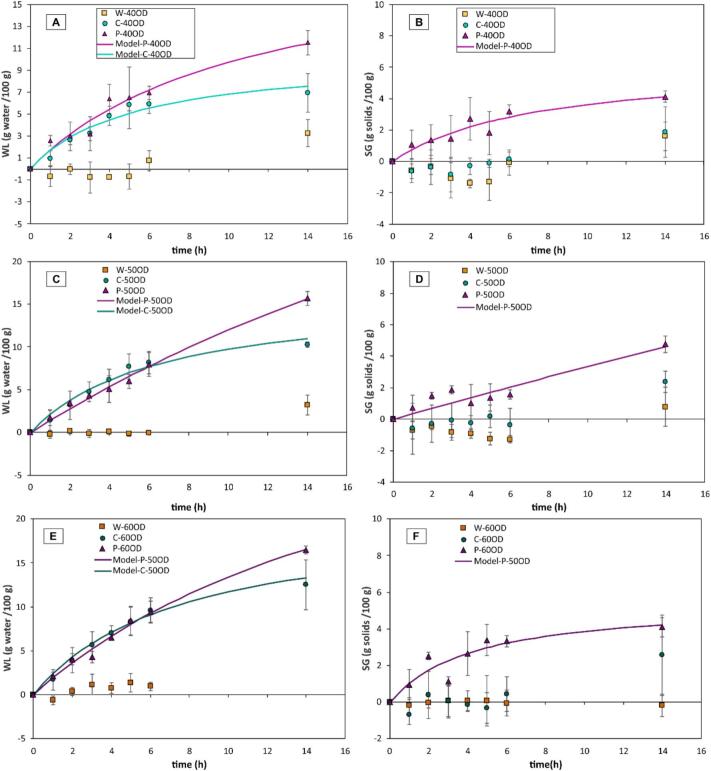
Water loss (A, C, E) and solids gain (B, D, F) under different conditions of OD (40, 50 and 60°Brix) applied to MM (C and P) samples. Dots indicate experimental data, the vertical bars represent the standard deviation (n = 4), and the continuous curves represent the data calculated according to the Peleg model (equations (3), (4).

Fig. 2 displays water loss (WL) (Fig. 2.A, C, and E) and solids gain (SG) (Fig. 2.B, D, and E) during OD at 40, 50, and 60°Brix of the mechanically modified blueberries. The Peleg model was fitted to the experimental data for SG and WL. A better fit was observed for the WL data, with R2 ≥ 0.838, while the fit for the SG data was lower (R2 ≥ 0.505). Specifically, solids gain (SG) was found to progress at a slower rate and commenced later in the process compared to water loss (WL). This lag in SG relative to WL influenced the model's ability to accurately represent the experimental results for each parameter, resulting in variations in the goodness of fit between the two mass transfer phenomena. To improve the mathematical fit of the SG during OD, it is recommended to evaluate OD kinetics for a longer period than that studied here.

The values of the Peleg model parameters *k_1_* and *k*_2_ for WL and SG are provided in Table 1 and Table 2, respectively. It was observed that in the whole blueberries (W), mass transfer did not occur at any OD level, whereas the pre-treatments of cut (C) and perforation (P) promoted WL during OD. The parameters of the Peleg model suggested that WL occurred more rapidly in the cut samples (C) at 50 and 60°Brix, reflected by lower *k_1_* values (Table 1). However, the equilibrium WL content was greater in the perforated samples, as indicated by lower *k*_2_ values (Table 1), which can also be visually confirmed in Fig. 2. Regarding SG, it was observed that only the P pre-treatment enabled the entry of solids from the osmotic solution into the blueberries, at all levels of OD (Fig. 2, Table 2).

**Table 1:** Water loss after 6 h of OD (average ± standard deviation) and parameters of the Peleg model [with 95% confidence interval] (equation (3) used to describe the water loss during OD. Uppercase letters represent mean comparisons among OD factor levels, and lowercase letters among MM factor levels, according to Tukey’s test (n = 3, p < 0.05). (*) means significant difference between 50OD and 50OD + US pre-treatments.

**Effect of MM and OD**			
**Treatment**	**Water loss** **after 6 h** **(g water/100 g)**	$k_{1}^{w}$**[CI 95%]** **(WL^−1^∙ h)**	$k_{2}^{w}$**[CI 95%]** **(WL^−1^)**
W-40OD	0.783 ± 0.914^Ab^	−	−
C-40OD	5.927 ± 0.870^Ba^	0.507 [0.368–0.646]	0.095 [0.069–0.121]
P-40OD	6.926 ± 0.591^Aa^	0.539 [0.371–0.707]	0.049 [0.025–0.072]
W-50OD	−0.074 ± 0.208^Ab^	−	−
C-50OD	8.172 ± 1.335^ABa^	0.408 [0.312–0.505]	0.062 [0.046–0.078]
P-50OD	7.912 ± 1.391^Aa^	0.690 [0.592–0.788]	0.015 [0.006–0.024]
W-60OD	0.947 ± 0.509^Ab^	−	−
C-60OD	9.617 ± 1.383^Aa^	0.370 [0.291–0.450]	0.048 [0.035–0.062]
P-60OD	9.405 ± 1.243^Aa^	0.503 [0.438–0.568]	0.024 [0.017–0.032]
**Effect of US-assisted OD**			
**Treatment**	**Water loss** **after 6 h** **(g water/100 g)**	$k_{1}^{w}$**[CI 95%]** **(WL^−1^∙ h)**	$k_{2}^{w}$**[CI 95%]** **(WL^−1^)**
W-50OD + US	1.777 ± 0.538*	1.635 [0.003–3.267]	0.303 [-0.077–0.684]
C-50OD + US	9.471 ± 2.336	0.483 [0.257–0.708]	0.025 [-0.022–0.073]
P-50OD + US	8.488 ± 0.915	0.447 [0.290–0.604]	0.045 [0.011–0.080]

**Table 2:** Solids gain after 6 h of OD (average ± standard deviation) and parameters of the Peleg model [with 95% confidence interval] (equation (4) used to describe the solids gain during OD. Uppercase letters represent mean comparisons among OD factor levels, and lowercase letters among MM factor levels, according to Tukey’s test (n = 3, p < 0.05). (*) means significant difference between 50OD and 50OD + US pre-treatments.

**Effect of MM and OD**			
**Treatment**	**Solids gain** **after 6 h** **(g solids/100 g)**	$k_{1}^{S}$**[CI 95%]** **(SG^-1^∙ h)**	$k_{2}^{S}$**[CI 95%]** **(SG^-1^)**
W-40OD	−0.076 ± 0.823^Ab^	−	−
C-40OD	−0.004 ± 0.494^Ab^	−	−
P-40OD	3.199 ± 0.387^Aa^	1.184 [0.437–1.931]	0.158 [0.042–0.275]
W-50OD	−1.287 ± 0.233^Ab^	−	−
C-50OD	−0.387 ± 1.052^Ab^	−	−
P-50OD	1.569 ± 0.304^Aa^	2.874 [1.631–4.118]	0.010 [-0.096–0.116]
W-60OD	−0.277 ± 0.704^Ab^	−	−
C-60OD	0.435 ± 0.933^Ab^	−	−
P-60OD	3.32 ± 0.669^Aa^	0.781 [0.341–1.220]	0.182 [0.100–0.264]
**Effect of US-assisted OD**			
**Treatment**	**Solids gain** **after 6 h** **(g solids/100 g)**	$k_{1}^{S}$**[CI 95%]** **(SG^-1^∙ h)**	$k_{2}^{S}$**[CI 95%]** **(SG^-1^)**
W-50OD + US	0.256 ± 0.562*	−	−
C-50OD + US	0.438 ± 2.118	−	−
P-50OD + US	2.167 ± 0.421	0.697 [-0.226–1.620]	0.318 [0.069–0.567]

Additionally, it is important to note that the pre-treatment P-50OD, in both the kinetics of WL and SG, showed a continuous increase with no evident trend toward equilibrium after 14 h of OD (Fig. 2). This indicates that, compared to the other pre-treatments, the P-50OD treatment would allow for greater WL and SG over prolonged OD times, as evidenced by the lower *k*_2_ values (Table 1 and Table 2).

**Fig. 3:**
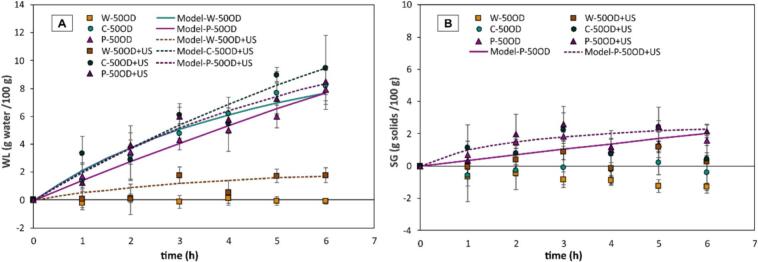
Water loss (A) and solids gain (B) under US-assisted OD (50°Brix) applied to MM (C and P) samples. The dots indicate experimental data, the vertical bars represent the standard deviation (n = 4), and the continuous curves represent the data calculated according to the Peleg model (equations (3), (4).

Fig. 3 shows WL (Fig. 3.A) and SG (Fig. 3.B) during OD at 50°Brix assisted with ultrasound (US), for whole samples (W) and mechanically modified blueberries. It was observed that in whole blueberries (W-50OD + US) and in pre-treated samples involving cutting (C-50OD + US) and perforation (P-50OD + US), ultrasound promoted WL compared to treatments without ultrasound (W-50OD, C-50OD and P-50OD). On the other hand, regarding SG during OD, ultrasound slightly enhanced solid uptake; however, considerable variation was observed in the results.

For statistical comparison purposes, the values obtained after 6 h of OD were considered; the results of WL and SG are shown in Table 1 and Table 2, respectively. Regarding the effect of the MM level, it was observed that both cut (C) and perforated (P) blueberries significantly increased the amount of WL at all OD levels compared to whole blueberries (W). However, across all OD concentrations, only the pre-treatment P had a significant effect on increasing solids gain compared to the W and C pre-treatment. On the other hand, with respect to the OD level, in whole blueberries (W), none of the OD concentrations had a significant effect on WL or solids gain. In cut blueberries (C), the OD level did not significantly affect SG; however, the amount of WL increased as the OD concentration increased, being higher at 50 and 60°Brix. In perforated blueberries (P), none of the OD levels had a significant effect on WL or SG. To evaluate the effect of OD at 50°Brix assisted by ultrasound, the results were compared with those from OD at 50°Brix without ultrasound. The application of US-assisted OD resulted in a significant increase in both SG and WL in whole samples (W) without mechanical pre-treatment (Table 1 and Table 2). However, when ultrasound was applied to mechanically modified samples (P and C), the effect was not statistically significant (*p > 0.05*), although a slight upward trend in SG and WL was observed.

Regarding the primary ultrasound mechanisms that improved mass transfer during OD of blueberries are: (i) Acoustic cavitation: the microbubbles implosion in the liquid medium generates localized microjets and shockwaves. When these occur near to blueberry surface could induce the detachment of the blueberry waxy layer and the possible formation of microchannels in the blueberry peel through acoustic cavitation, thereby accelerating water removal and solids uptake [38], [39]. (ii) Sponge effect: Ultrasound-induced pressure oscillations could expel water from the interior while allowing osmotic solutes to penetrate. (iii) Cell disruption and increased permeability: Cavitation and microstreaming damage cell membranes and cell walls, increasing tissue permeability. This was evidenced by the migration of anthocyanin pigments from the blueberry skin into the mesocarp (Fig. 6), confirming membrane disruption. (iv) Boundary layer reduction: Ultrasound agitation reduced the external mass transfer resistance at the liquid–solid interface, enhancing concentration gradients.

However, the improvement in mass transfer during OD was mainly attributed to the MM, which already created pathways for water release and solids uptake overshadowing any additional effects that could be attributed to ultrasound treatment. Overall, the results demonstrated that the OD process of blueberries is a slow process requiring prolonged times. However, mass transfer rates could be improved with mechanical pre-treatments. Similar findings regarding improved mass transfer during OD were reported by Yu, Jin, Fan and Xu [14] and Yu, Jin, Fan and Wu [13] in blueberries pretreated with pulsed electric fields (PEF), where OD times were reduced by more than 50% compared to whole blueberries. However, those studies employed prolonged OD times (70°Brix and 40°C), requiring more than 96 h to reach the desired OD levels.

### 3.2 Pre-treatment effect on convective drying

**Fig. 4:**
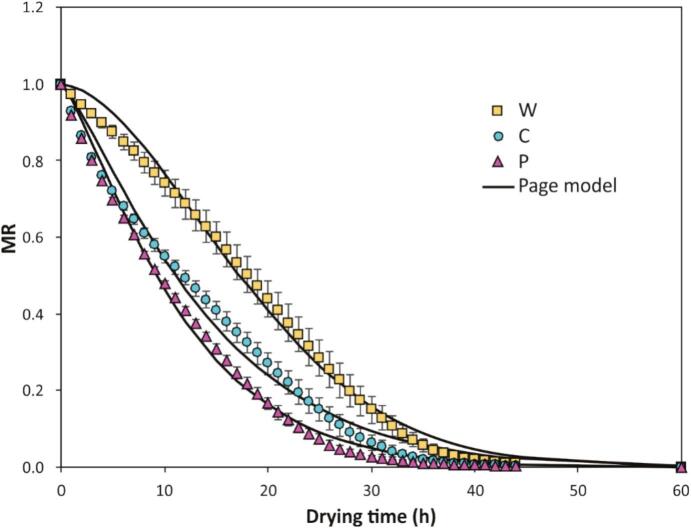
Convective drying kinetics of control (whole, W) and mechanically modified samples (C and P). The dots represent the average of experimental data, the vertical bars the standard deviation (n = 3), and the continuous curves represent data modeled according to the Page model.

**Fig. 5:**
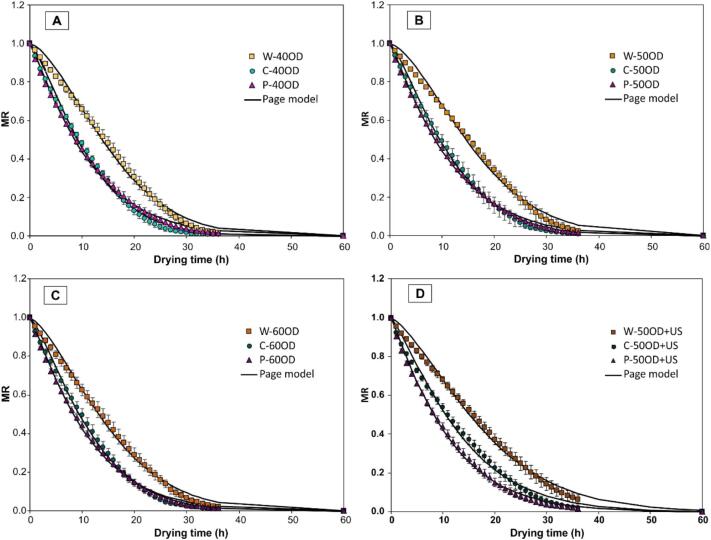
Effect of OD at (A) 40°Brix (40OD), (B) 50°Brix (50OD), (C) 60°Brix (60OD), and (D) 50°Brix assisted by ultrasound (50OD + US) on the drying kinetics for each level of MM. The dots represent the average of experimental data, the vertical bars the standard deviation (n = 3), and the continuous curves correspond to data modeled according to the Page model.

To assess the effect of pre-treatments on convective drying, drying kinetics were constructed for blueberries subjected to MM (Fig. 4) and for blueberries treated with MM followed by OD (Fig. 5). These figures illustrate that blueberry drying is a prolonged process: under the studied conditions, whole blueberries required 52 h to reach a final moisture content of 12% w.b. (or 0.136 g water/g dry matter). The reported drying times are consistent with previous studies where blueberry drying at 60°C took 43 h [8], [14]. This duration is mainly due to the blueberry skin, which acts as a barrier to moisture removal [18], particularly during the initial drying phase. Therefore, pre-treatments targeting the blueberry skin facilitate water vapor migration from the interior to the drying environment.

Fig. 4 shows that, compared to untreated whole blueberries (W), cut (C) and perforated (P) blueberries displayed more rapid exponential decreases in their drying curves. Regarding the combination of MM and OD (Fig. 5), all OD levels demonstrated similar drying behavior for treatments C and P, which differed from that of whole blueberries (E) (Fig. 5.A, 5.B, and 5.C). However, when ultrasound-assisted OD was applied (Fig. 5.D), a marked differentiation in drying kinetics was observed among MM levels. The drying behavior was described using the Page model, with its parameters (*k* and *n*) presented in Table 3. The parameter *k* represents the drying rate during the initial stage. For the effect of MM, it was found that, at every OD level, the perforated treatment (P) exhibited the highest drying rate compared to whole blueberries (W). Conversely, no significant differences in drying rate were observed across OD application levels for whole (W) and cut (C) blueberries. However, OD at 40, 50, or 60°Brix significantly increased the drying rate for perforated blueberries (P-40OD, P-50OD, P-60OD). Regarding US-assisted OD, a significant increase in drying rate was noted only for perforated blueberries (P) with ultrasound-assisted OD at 50°Brix, with a positive trend also visible for other MM levels.

**Table 3:** Parameters (average ± standard deviation) of the Page model (equation (6) describing convective drying kinetics as a function of MM and OD. Different uppercase letters indicate significant differences between OD levels, and different lowercase letters indicate significant differences between MM levels, according to Tukey’s test (n = 3, α = 0.05). (*) means significant difference between 50OD and 50OD + US pretreatments.

**Effect of MM and OD**			
**Treatment**	***k* (h^-n^)**	***n* (−)**	***R^2^***
**W**	0.005 ± 0.002^Ab^	1.769 ± 0.090^Aa^	≥ 0.993
**C**	0.036 ± 0.002^Aa^	1.227 ± 0.011^Ab^	≥ 0.987
**P**	0.044 ± 0.001^Ba^	1.239 ± 0.017^Ab^	≥ 0.996
**W-40OD**	0.011 ± 0.002^Ac^	1.594 ± 0.040^Ba^	≥ 0.993
**C-40OD**	0.036 ± 0.007^Ab^	1.343 ± 0.071^Ab^	≥ 0.995
**P-40OD**	0.058 ± 0.002^Aa^	1.154 ± 0.025^Ac^	≥ 0.996
**W-50OD**	0.010 ± 0.001^Ac^	1.574 ± 0.064^Ba^	≥ 0.992
**C-50OD**	0.035 ± 0.007^Ab^	1.323 ± 0.039^Ab^	≥ 0.995
**P-50OD**	0.059 ± 0.005^Aa^	1.145 ± 0.020^Ac^	≥ 0.997
**W-60OD**	0.016 ± 0.005^Ac^	1.482 ± 0.086^Bab^	≥ 0.993
**C-60OD**	0.034 ± 0.006^Ab^	1.342 ± 0.041^Ab^	≥ 0.996
**P-60OD**	0.060 ± 0.005^Aa^	1.154 ± 0.024^Ac^	≥ 0.997
**Effect of US-assisted OD**			
**Treatment**	***k* (h^-n^)**	***n* (−)**	**R^2^**
**W-50OD + US**	0.015 ± 0.002*	1.416 ± 0.067*	≥ 0.993
**C-50OD + US**	0.039 ± 0.003	1.225 ± 0.003*	≥ 0.993
**P-50OD + US**	0.067 ± 0.005	1.111 ± 0.025	≥ 0.997

All drying times reported were estimated to reach the same target moisture content of 12% w.b., calculated from the fitted Page model. Compared to untreated whole blueberries (W), drying time was reduced on average by 2.91% and 19.09% for cut (C) and perforated (P) treatments, respectively. These reductions are attributed to MM, decreasing skin resistance and allowing water to exit the berry faster. For the combined effect of MM and OD and US-assisted OD a 50°Brix, relative to untreated blueberries (W), drying time to reach 12% w.b. final moisture was reduced by an average of 13.66% (W-40OD), 32.26% (C-40OD), 18.37% (P-40OD), 9.02% (W-50OD), 28.50% (C-50OD), 20.81% (P-50OD), 13.17% (W-60OD), 30.03% (C-60OD), 24.64% (P-60OD), −9.57% (W-50OD + US), 15.82% (C-50OD + US), and 19% (P-50OD + US). It is important to note that of all the pre-treatments applied, pre-treatment W-50OD + US showed an increase in drying time compared to the control (W). This was probably because the solids gained in this treatment (as shown in Table 2) formed a surface layer (crust) that hindered water evaporation during drying, especially in the final drying stage. Conversely, in the samples with MM, despite the solids gain, there were sufficient pathways for water evaporation during drying.

Previous studies have reported reductions in blueberry drying times by applying pre-treatments or novel drying methods. Convective drying time has been reduced by 31.25% using plasma pre-treatments [40]; freeze-drying time decreased by 61.47% with 30–40 perforations per berry [17]; convective drying time reduced by 32.14% with vacuum pulses [41]; freeze-drying time reduced by 60.6% for blueberries cut in halves; convective drying time reduced by 23.5% for berries pretreated with nine CO_2_ laser perforations [18]; drying time reduced by 39.53% with a 1-minute alkaline emulsion pre-treatment [8]; and vacuum drying at 60°C reduced by 30% using pulsed electric fields [42]. As observed, in comparison with processes involving convective drying, this research achieved similar levels of drying time reduction by utilizing simpler pre-treatments and probably with lower energy consumption.

Consequently, the most effective treatments for reducing estimated drying time were C-40OD and C-60OD. Notably, these treatments were not the same as those with the highest drying rates (P-40OD, P-50OD, P-60OD). This suggests that perforation treatments accelerated the initial drying stage (higher *k* values), while cut treatments were more effective in reducing the final drying stage. Possibly, the channels formed by perforation facilitated water removal at the beginning of the process, but their effectiveness diminished as berry shrinkage increased the tortuosity of the channels with ongoing drying.

### 3.3 Viscoelastic properties

Table 4 shows the parameters of the generalized Maxwell model for every treatment. This model is demonstrated to be suitable for the relaxation test, as the majority of the R^2^ values exceed 0.95. Therefore, its parameters can be correlated to the structure of blueberries to explain its viscoelasticity.

**Table 4:** Maxwell model parameters (Equation (7) for every stress-relaxation curve of control (W-wholes) blueberries and pre-treated with MM (C and P), OD (40, 50 and 60°Brix), and US-assisted OD (50OD + US). The data is represented as average ± standard deviation. Superscript letters represent Tukey’s test (n = 20, α = 0.05), considering uppercase letters for comparing effect of OD and lowercase letters for comparing the effect of MM. (*) means significant difference between 50OD and 50OD + US pretreatments.

**Effect of MM and OD**									
T	***ɛ***	***t_1_* (s)**	***t_2_* (s)**	***ξ_e_* (kPa)**	***ξ_1_* (kPa)**	***ξ_2_* (kPa)**	***ƞ_1_* (kPa∙s)**	***ƞ_2_* (kPa∙s)**	**R^2^**
**W**	0.08 ± 0.008	10.26 ± 0.26^Ab^	0.66 ± 0.04^Aa^	51.67 ± 9.24^Ab^	14.44 ± 2.43^Ab^	14.42 ± 2.61^Ab^	148.53 ± 27.26^Ab^	9.61 ± 1.99^Ab^	≥0.997
**C**	0.082 ± 0.01	10.90 ± 0.26^Aa^	0.71 ± 0.02^Ba^	62.29 ± 13.52^Aa^	18.35 ± 3.88^Aa^	17.89 ± 4.43^Aa^	199.51 ± 40.35^Aa^	12.75 ± 3.03^Aa^	≥0.997
**P**	0.073 ± 0.008	11.26 ± 0.308^Ba^	0.71 ± 0.03^Ca^	39.42 ± 6.19^Ac^	11.59 ± 2.76^Ac^	11.91 ± 1.45^Ac^	130.12 ± 29.15^Ab^	8.44 ± 1.29Ab	≥0.997
**W-40OD**	0.083 ± 0.013	10.34 ± 0.46^Ab^	0.68 ± 0.04^Ab^	48.36 ± 8.49^Aa^	13.91 ± 2.17^Aa^	13.79 ± 2.59^Aa^	143.84 ± 20.35^Aa^	9.35 ± 1.69^Aa^	≥0.997
**C-40OD**	0.082 ± 0.009	11.12 ± 0.66^Aa^	0.79 ± 0.13^Aa^	15.34 ± 5.47^Cb^	5.73 ± 1.87^Cb^	4.68 ± 1.75^Cb^	63.59 ± 21.28^Cb^	3.71 ± 1.43^Cb^	≥0.995
**P-40OD**	0.086 ± 0.011	11.68 ± 0.17^ABa^	0.79 ± 0.03^Ba^	14.96 ± 3.84^Bb^	5.23 ± 1.01^Bb^	4.98 ± 0.41^Bb^	60.94 ± 11.03^Bb^	3.94 ± 0.47^Bb^	≥0.996
**W-50OD**	0.082 ± 0.017	10.52 ± 0.44^Aa^	0.68 ± 0.05^Ab^	40.87 ± 8.42^Aa^	12.56 ± 3.02^Aa^	11.78 ± 3.01^Aa^	133.09 ± 35.38^Aa^	8.03 ± 2.24^Aa^	≥0.996
**C-50OD**	0.083 ± 0.009	10.99 ± 0.79^Aa^	0.78 ± 0.13^ABa^	27.63 ± 7.48^Bb^	9.90 ± 2.69^Ba^	8.2 ± 2.62^Bb^	108.79 ± 30.06^Ba^	6.29 ± 1.91^Ba^	≥0.994
**P-50OD**	0.088 ± 0.008	10.55 ± 0.56^Ca^	0.86 ± 0.03^ABa^	14.98 ± 3.39^Bc^	5.91 ± 1.02^Bb^	4.32 ± 0.44^Bc^	62.05 ± 9.15^Bb^	3.71 ± 0.51^Bb^	≥0.993
**W-60OD**	0.081 ± 0.008	10.05 ± 1.52^Ac^	0.73 ± 0.11^Aa^	45.39 ± 6.62^Aa^	14.29 ± 2.28^Aa^	11.27 ± 4.02^Aa^	142.19 ± 25.79^Aa^	7.96 ± 2.43^Aa^	≥0.974
**C-60OD**	0.087 ± 0.011	10.99 ± 0.77^Ab^	0.80 ± 0.13^Aa^	27.01 ± 8.79^Bb^	10.26 ± 3.46^Bb^	8.20 ± 2.84^Bb^	112.54 ± 37.44^Bb^	6.54 ± 2.21^Bb^	≥0.987
**P-60OD**	0.092 ± 0.011	12.05 ± 0.27^Aa^	0.92 ± 0.03^Ab^	9.89 ± 2.01^Bc^	3.90 ± 0.50^Bc^	3.30 ± 0.25^Bc^	46.91 ± 5.37^Bb^	3.03 ± 0.32^Bb^	≥0.995
**Effect of US-assisted OD**									
**T**	***ɛ***	***t_1_* (s)**	***t_2_* (s)**	***ξ_e_* (kPa)**	***ξ_1_* (kPa)**	***ξ_2_* (kPa)**	***ƞ_1_* (kPa∙s)**	***ƞ_2_* (kPa∙s)**	**R^2^**
**W-50OD + US**	0.072 ± 0.008	10.77 ± 0.43	0.73 ± 0.04*	49.19 ± 10.31*	14.27 ± 2.61	13.66 ± 2.86	153.43 ± 27.58	9.92 ± 1.91*	≥0.997
**C-50OD + US**	0.072 ± 0.005	11.51 ± 0.34*	0.86 ± 0.04*	13.85 ± 4.35*	5.33 ± 1.56*	4.42 ± 1.31*	61.24 ± 17.95*	3.76 ± 1.03*	≥0.994
**P-50OD + US**	0.072 ± 0.005	11.12 ± 0.54*	0.79 ± 0.09*	10.86 ± 2.39*	4.42 ± 1.012*	3.61 ± 0.68*	49.17 ± 12.02*	2.84 ^±^ 0.67*	≥0.952

**Fig. 6:**
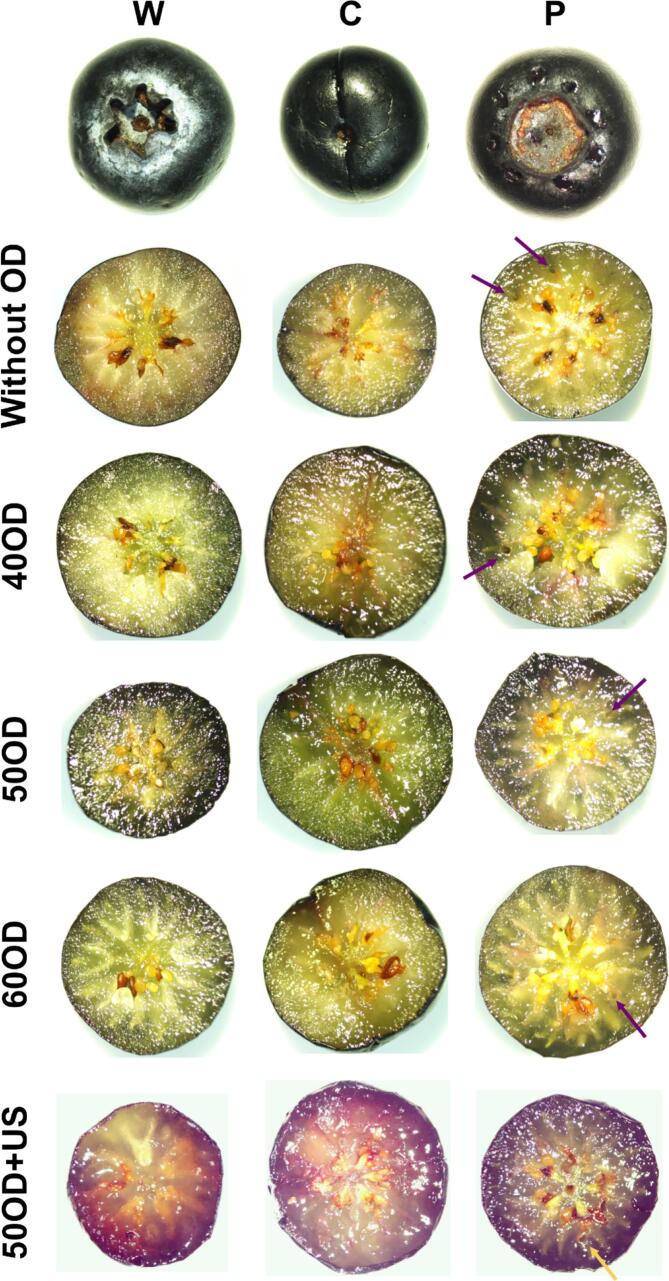
Photographs of blueberries under different treatments. Every berry was transversely cut. Arrows point out some visible holes caused by perforation.

**Fig. 7:**
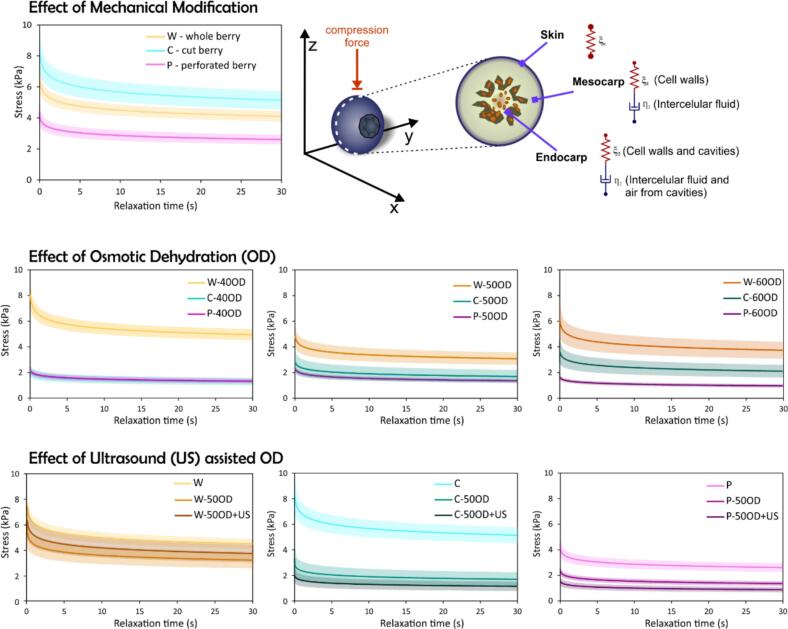
Stress-relaxation curves of whole and pre-treated blueberries under different conditions: MM (C and P), OD (40, 50 and 60°Brix) and US-assisted OD (50°Brix). The lines represent the average of experimental data, and the shaded regions represent the confidence bands (95% of confidence).

Blueberry structure presents a solid matrix structured by cells of the epicarp, mesocarp and pericarp, and fluids such as water and trapped gases [43] (Fig. 6 and Fig. 7). Both solids and fluids give viscoelastic behavior to the blueberry. Therefore, the spring elements from Maxwell model can be related to the solid structure of the berry, which presents mostly elastic behavior. On the other hand, the dashpot elements would explain the viscous behaviors of the berry’s fluids. This analysis was similarly performed in studies for yellow melon cylinders [44] and pumpkin cylinders [23].

Following this procedure, the elastic elements *ξe*, *ξ_1_* and *ξ_2_* can be related to solid structures and viscous elements *ƞ_1_* and *ƞ_2_* to fluids. Firstly, *ξe* may be associated with the skin of the berry, as this parameter is not connected to a viscous element, and the solid structure of the peel contains negligible fluids. *ξ_1_* and *ƞ_1_* could be associated with mesocarp tissue, where the solid component would be the cell wall and the fluid component would be cytoplasm and any other intracellular fluid. Finally, *ξ_2_* and *ƞ_2_* may be associated with endocarp, where the solid component would be cell walls and seeds, while the viscous component would be represented by intercellular and extracellular fluids. In this case, more gases would be presented since there are cavities where seeds are attached (Fig. 7).

Regarding the Generalized Maxwel model parameters, for the whole berry, the value of *ξe* is higher than *ξ_1_* and *ξ_2_* (Table 4). This makes sense, since the skin is more elastic and resistant than the mesocarp and endocarp tissue. On the other hand, *ƞ_1_* values is higher than *ƞ_2_* value, which means that viscosity of the fluids in mesocarp tissue may be higher than the fluids from endocarp. In fact, endocarp presents more voids filled with air (Fig. 6) than the mesocarp. This could explain lower value of viscosity (*ƞ_2_*).

When MM pre-treatment was performed to berries, the viscoelastic parameters change, especially when they are perforated (Table 4). Cut berries presented similar viscoelastic properties than the whole berry. However, the elasticity and viscosity of perforated berries drastically decay. For instance, the elasticity related to the skin reduced by around 24%. Other work also reported texture change by puncture, where maximum force of compression was reduced around 60% in blueberries after performing 30–40 punctures of 0.5 mm of diameter [45]. In fact, the skin structure was weakened by perforation, causing the reduction of its elasticity parameter.

In the case of OD pre-treatment, Fig. 7 indicates that perforated berries experienced the most significant change in maximum stress (related to maximum compression force) when using the three different osmotic solutions, followed by cut berries and whole berries. Osmotic dehydration creates a hyperosmotic environment outside the cell. Water flows out through osmosis via the plasma membrane and cell wall, causing cell plasmolysis (shrinkage and separation of the plasma membrane from the cell wall). The resulting loss of cell turgor reduces the elastic and viscous components of the viscoelastic structure. Therefore, it was anticipated that the whole berry would be the least affected by osmotic pre-treatment, as the skin serves as a barrier for mass transfer. These changes in texture can be related to the pectinase enzyme activity, which is more available due to cell membrane damage by osmotic solutions, contributing to softening and reduced elasticity [46].

Concerning Maxwell model parameters, elasticity and viscosity values slightly reduced when OD was performed on whole berries (Table 4). In this case, the parameters were significantly reduced when 50°Brix osmotic solution was used. This reduction may be related to the WL (Fig. 3) [47]. Although the WL was slightly higher at 60°Brix, the gain in solids was significantly greater at 60°Brix than at 50°Brix. This may have compensated for the loss of firmness in the blueberry by increasing its solid content.

For cut berries, both the elastic and viscosity elements were reduced when the 40°Brix solution was used. For 50°Brix and 60°Brix, the reduction was significant but not as much as for 40°Brix. This may be due to crust formation at higher osmotic solution concentrations, creating a seal on the cut section. On the other hand, perforated berries showed the same response to all osmotic solution concentrations, with elasticity and viscosity values reduced similarly to dehydrated cut berries at 40°Brix. Apparently, the holes were more difficult to block by a higher osmotic solution concentration.

In general, OD has been demonstrated to reduce viscoelastic properties, such as in melon [48], apple and banana [49]. In contrast to other dehydration processes such as air convective drying, OD acts as a plasticizer, reducing sample rigidity. This is because the process does not drastically reduce water activity and adds solutes as sucrose. On the other hand, dehydration by hot air decreased water mobility, which caused an increase of rigidity of sample [50].

Regarding the use of ultrasound, it exerts additional and more severe effects on the cellular structure compared to OD alone and significantly reduced all the elasticity and viscosity values for cut and perforated berries. This reduction is likely due to damage to the cut section and the holes on perforated berries debilitating the structure, as ultrasound did not notably affect WL or SG (Table 1 and Table 2). In other words, texture change may be caused by structure modification and not by dehydration. This can be observed in Fig. 6, where diffusion of pigments can be appreciated caused by ultrasound. Anthocyanin pigments (primarily located in the vacuoles of skin cells) migrate into the mesocarp tissue under US treatment, indicating tonoplast and membrane rupture. This intracellular disruption also partially explains the enhanced extractability of bioactive compounds observed in US-treated samples before drying.

The pigments from the skin diffused to mesocarp which led to color change. This happened for every treatment, suggesting cell damage.

On the other hand, for whole berries, the elasticity of the skin (*ξ_e_*) and the viscosity of the endocarp (*ƞ*_2_) increased. This is probably because ultrasound accelerates mass transfer, allowing sucrose to enter the berries, but mainly in the region between the skin and the mesocarp. Consequently, these additional solids increased the skin’s elasticity. In fact, the *SG* during OD was significantly higher when ultrasound was applied (Table 2). Concerning endocarp viscosity, ultrasound may have damaged cells near the air voids where the seeds are located, releasing cytoplasm and filling the voids with a more viscous fluid instead of trapped gases. This structural damage is also evident in Fig. 6, where diffused pigments can be observed.

#### 3.3.1 Relation between viscoelastic properties and mass transfer parameters

**Fig. 8:**
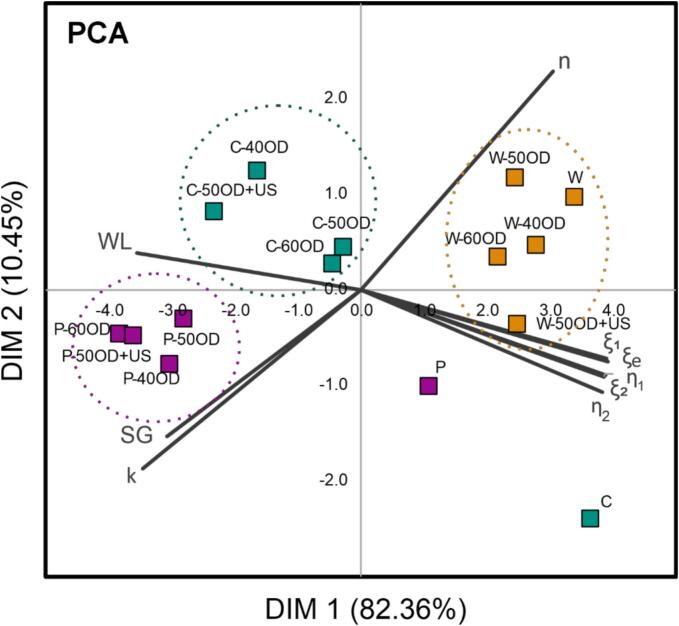
Principal Component Analysis (PCA) which associates viscoelastic properties (Maxwell model parameters), drying kinetics (Page model parameters) and OD (WL and solid sained) regarding different OD pre-treatments. Square represents the pre-treatments and vectors the correlation among the properties.

Fig. 8 shows the Principal Component Analysis applied to the viscoelastic properties, drying and OD kinetics comparing the different pretreatments. The combination of dimensions 1 and 2 (DIM 1 and DIM2) described 92.81% of the considered response variables, considering the highest explained variance. PCA differentiated three groups with similar OD-pretreatments regarding viscoelastic, drying and OD characteristics. One group corresponds to all pre-treatments where whole berry (W) was used. This indicates that despite the applied OD level, whole berries were not significantly affected by this pre-treatment. On the other hand, other two groups were formed: one by OD-pretreated cut berries and other by OD-pretreated perforated berries. Nevertheless, the cut and perforated berries without OD pre-treatment were not grouped, even localized far from their groups. This match with the result of OD-pretreatment significantly affected cut and perforated berries, specifically regarding WL and SG.

In addition, PCA helps identify the relationships among viscoelastic properties, drying kinetics, and OD results. The vectors representing the Maxwell model parameters are in the opposite quadrant to that of WL, indicating an inverse relationship, where the greater WL the lower viscoelastic parameters. This supports the idea that as cells lose water, they become less elastic, as shown in Fig. 7. Indeed, OD caused the relaxation curves to shift markedly downward compared with those without pre-treatment, confirming a loss of elasticity.

Further, PCA also indicates that Page parameter (*k*) from drying kinetics did not correlate with viscoelastic properties. This suggests that the change on viscoelastic properties of berries was not enough to affect the drying kinetics. On the other hand, the quantity of SG during OD, correlated to the Page’s model parameter *k*. This suggests that OD-pretreatments that caused more SG, accelerated the initial stage of blueberry drying.

### 3.4 Rehydration

**Fig. 9:**
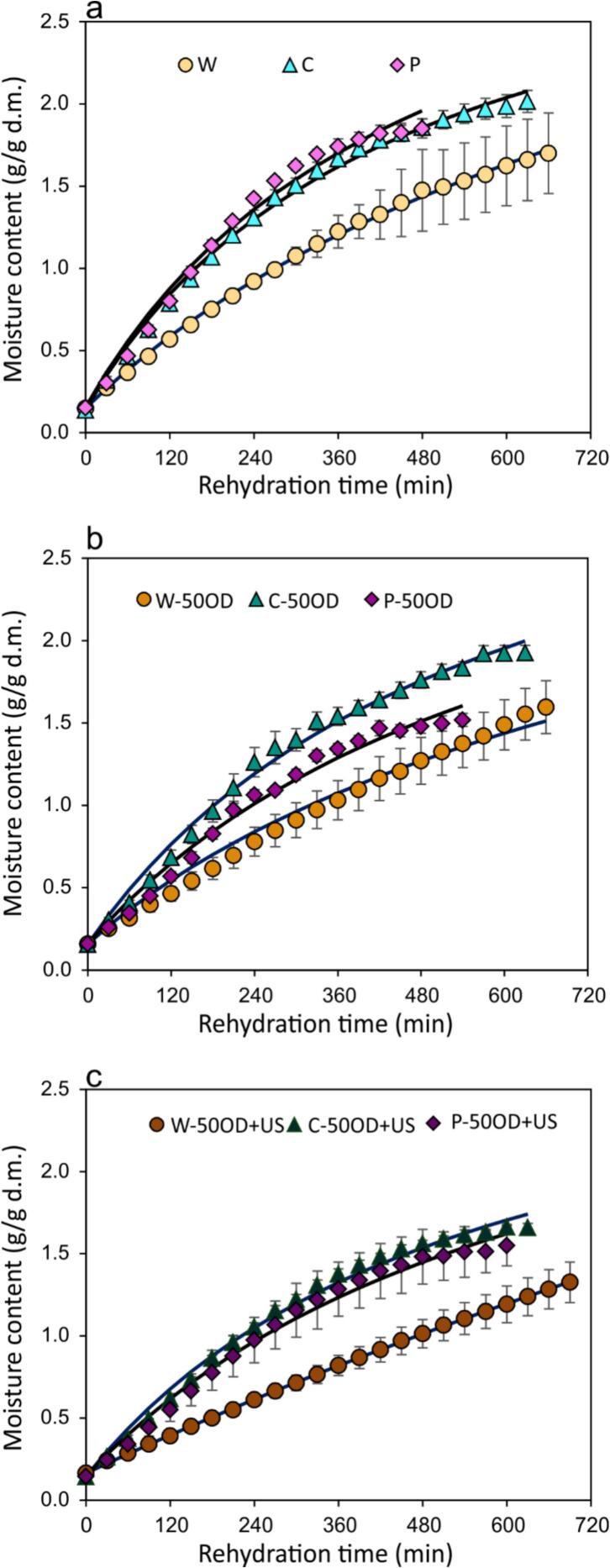
Rehydration curves of dried blueberries at different treatments: a. MM effect; b. OD pre-treatment effect; c. US-assisted OD effect. The dots represent the average of the experimental data, vertical bars are the standard deviation, and the continuous lines represent the Peleg model.

Fig. 9 presents the rehydration rates of dried blueberries. Perforated and cut berries absorbed water faster than whole berries due to holes that facilitated water entry. With OD (50°Brix), cut berries showed the fastest rehydration, likely due to gaps (cut section) allowing easier water entry. In perforated berries, hole sealing by solids during drying may have slowed rehydration. However, with ultrasound application during OD, both cut and perforated berries rehydrated at similar rates, probably because ultrasound-induced cell damage enlarging the gaps from cutting and puncturing.

Whole berries rehydrated more slowly than mechanically modified ones, regardless of pre-treatment, due to the epicarp acting as a barrier. Nevertheless, whole berries treated with ultrasound and OD show even slower rehydration than untreated or “in natura” samples, as indicated by the nearly linear rehydration curve in Fig. 8, pointing to a prolonged process. This could be unexpected for most of vegetable samples [51], but it might be caused by crust formation between the epicarp and mesocarp due to the additional SG caused by ultrasound.

Rehydration curves were suitably fitted to Peleg’s models, whose parameters are presented in Table 5. Regarding *k_1_* parameters (inverse of rehydration rate), W-50OD + US samples presented almost double the values compared to W-50OD and E. This agrees with the curve’s behavior explained in the previous paragraph. For the cut and perforated berries, OD and OD + US treatments did not significantly change *k_1_* parameter. Finally, concerning *k_2_* parameter (related to the inverse of equilibrium moisture content), this did not significantly change with any treatment. However, it is important to mention that in long rehydration processes not only water is gained, but also solids are significantly lost in water [52]. Therefore, the analysis of rehydration by mass balance could not give us exact results for equilibrium moisture content.

**Table 5:** Peleg’s model parameters (Equation (10) from rehydration modeling of dried blueberries. Superscript letter represents Tukey’s mean comparison test (n = 3, α = 0.05): Upper-case letters compare among osmotic pre-treatment (without, 50OD and 50OD + US) and lower-case letters compare among W (whole), C (cut) and P (perforated).

Treatment	*k_1_* (min · d.b.^-1^)	*k_2_* (d.b.^-1^)	R^2^
W	241.0 ± 29.4^Ba^	0.285 ± 0.17^Aa^	≥ 0.993
C	134.8 ± 7.4^Ab^	0.302 ± 0.011^Aa^	≥ 0.995
P	133.0 ± 8.7^Ab^	0.277 ± 0.033^Aa^	≥ 0.986
W-50OD	281.1 ± 91.2^Ba^	0.32 ± 0.192^Aa^	≥ 0.952
C-50OD	164.9 ± 20.8^Aab^	0.282 ± 0.03^Aa^	≥ 0.992
P-50OD	210.1 ± 13.7^Ab^	0.304 ± 0.017^Aa^	≥ 0.983
W-50OD + US	505.5 ± 37^Aa^	0.133 ± 0.062^Aa^	≥ 0.999
C-50OD + US	185.7 ± 13.8^Ab^	0.331 ± 0.017^Aa^	≥ 0.991
P-50OD + US	222.0 ± 44.5^Ab^	0.311 ± 0.011^Aa^	≥ 0.986

Dried blueberries are most likely intended for direct consumption or as ingredients in other dry products such as pastries, energy bars and mixed with cereals. Consequently, rehydration analysis is not commonly emphasized in these processing contexts. However, understanding the rate at which the berries rehydrate can provide information about consumer perception. For instance, increased saliva penetration during mastication could enhance flavor release and intensity. In addition, the rehydration rate affects how fast blueberries can be reconstituted on breakfast morning cereals, when they are mixed with milk or yogurt.

### 3.5 Bioactive compounds and antioxidant capacity

The total phenolic compounds (TPC), total monomeric anthocyanins (TMA), and antioxidant capacity (AC) levels in fresh blueberries (W, whole blueberries) were 22.11 ± 0.76 mg GAE/g d.m, 8.37 ± 0.62 mg cya-3-glu/g d.m, and 44.67 ± 1.94 mg Trolox/g d.m, respectively. Similar values for TPC and anthocyanins in fresh blueberries were reported by Liu, Xie, Zielinska, Pan, Wang, Deng, Wang and Xiao [41]. In contrast, these levels were higher than those reported by Yu, Jin and Xiao [42], likely due to the different variety and origin of the blueberries.

**Fig. 10:**
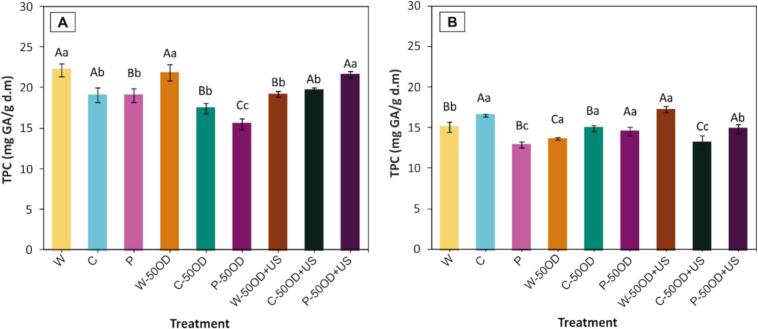
Total phenolic content before (A) and after drying (B). Letters represent the Tukey’s mean comparison test (n = 6, α = 0.05): Upper-case letters compare among OD pre-treatment (without, 50OD and 50OD + US) and lower-case letters compare among W (whole), C (cut) and P (perforated).

Fig. 10 illustrates the TPC after the different pre-treatments (Fig. 10.A) and after drying (Fig. 10.B). Regarding the pre-treatment effect on initial TPC (Fig. 10.A), a significant reduction in TPC was observed for cut and perforated blueberries when compared to whole (W), this was because MM increased the exposure of internal tissues, making bioactive compounds more susceptible to degradation. OD of whole blueberries (W-50OD) did not result in changes in TPC content; however, when ultrasound-assisted OD was applied (W-50OD + US), a decrease in TPC was observed. On the other hand, when OD pre-treatment was applied in cut (C-50OD) and perforated blueberries (P-50OD), a significant reduction in TPC occurred. This could have been due to the possible leaching of bioactive compounds during OD of the cut and perforated samples. Similar trends in the reduction of bioactive compounds have been reported following OD treatments (65°Brix × 5h at 30°C), with effects being amplified by the application of vacuum [12]. It has also been documented that the loss of bioactive compounds increases with WL during OD, likely due to biochemical degradation and the physical migration of compounds from berries into the osmotic solution [13], [14]. In contrast, when ultrasound-assisted OD was applied in these mechanically modified samples, TPC levels remained similar (C-50OD + US) or were even higher (P-50OD + US) than in the samples with only MM.

Therefore, the application of US-assisted OD pre-treatment enhanced the level of TPC in mechanically modified blueberries, especially in perforated (P-50OD + US) blueberries. A possibility is that ultrasound may have enhanced the extraction of TPC. When ultrasound is applied to a food matrix submerged in a liquid medium, mechanisms known as acoustic cavitation, the sponge effect, and microjets occur, leading to modifications in the food structure. These include, for example, cell wall disruption, plastid rupture, and dispersion of components within the cell. These structural changes influence the quantity of bioactive compounds in different ways, either increasing their quantity due to greater extractability of the components or decreasing their quantity due to their degradation caused by greater exposure to deteriorating conditions [53]. In fact, the application of ultrasound-assisted OD resulted in the migration of blueberry skin pigments into the mesocarp, as shown in Fig. 6, indicating cell disruption and pigment release. These improvements in extraction may have compensated for the losses due to degradation and/or leaching of total phenolic compounds observed in treatments involving cutting and perforation without ultrasound.

Compared to the initial TPC level (in whole blueberries before drying), the drying process led to a reduction in TPC across all treatments (Fig. 10.B), this was expected because the samples were exposed to deteriorating conditions during drying (mainly temperature and oxygen) decreasing the TPC, especially in the P and C-50OD + US treatments, which showed decreases of over 40%. In contrast, also regarding the initial TPC level in whole blueberries, the pre-treatments that best preserved phenolic content after drying were the C and W-50OD + US treatments, both with TPC reductions < 25%.

Additionally, to better understand the influence of pre-treatments on the preservation/degradation of TPC during drying, a comparison was made with their respective initial TPC content for each pre-treatment. The pre-treatments that best preserved the TPC were P-50OD, W-50OD + US, C, and C-50OD, with over 85% preservation of initial TPC. Possible factors promoting TPC preservation in these samples include the protective effect of OD [39] and the reduction in drying time achieved by applying the pre-treatments. On the other hand, the treatments with the highest level of TPC degradation during drying were W-50OD and C-50OD + US. In these cases, the prolonged drying time (W-50OD) and the greater exposure to deteriorating conditions, intensified by modifications in the structure with ultrasound (C-50OD + US), may have contributed to the issue.

**Fig. 11:**
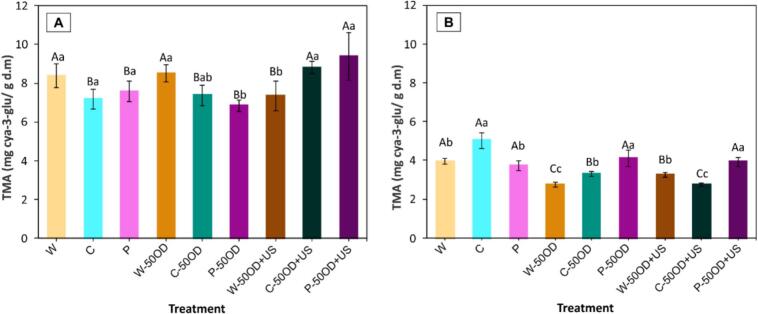
Total monomeric anthocyanins before (A) and after drying (B). Letters represent the Tukey’s mean comparison test (n = 6, α = 0.05): Upper-case letters compare among osmotic pre-treatment (without OD, 50OD and 50OD + US) and lower-case letters compare among W (whole), C (cut) and P (perforated).

Fig. 11 displays the total monomeric anthocyanin (TMA) content after the different pre-treatments (Fig. 11.A) and after drying (Fig. 11.B). Regarding the effect of pre-treatments, at all levels of OD, a decrease in total monomeric anthocyanins (TMA) was observed when MM (C and P) were applied without ultrasound assistance. This results in degradation rates between 9.5% and 18.5% respectively. In contrast, when ultrasound-assisted OD was applied to these mechanically modified samples (C-50OD + US and P-50OD + US), TMA levels increased up to 5% and 12% respectively. The increase in TMA content, similar to the trend observed for TPC in these samples, may be attributed to improved anthocyanin extraction resulting from structural changes induced by ultrasound treatment [54].

The drying process induced a reduction in TMA across all pre-treatments (Fig. 11.B), especially in the W-50OD and C-50OD + US treatments, both of which experienced a 67% decrease relative to the initial anthocyanin levels in fresh whole blueberries. In fact, anthocyanins in berries such as blueberries are highly susceptible to degradation during drying, especially during the final stage of the process, since at this point the moisture content is lower and the internal temperature is higher [55]. On the other hand, both in relation to the initial TMA content in whole samples without pre-treatment and to the initial TMA content in pre-treated samples, the treatments that best preserved anthocyanin content were C, P-50OD, and P-50OD + US. In these cases, anthocyanin levels remained above 4 mg cya-3-glu/g d.m, which is comparable to the values reported by Zhang, Zhang, Chen and Guan [56] for air-dried blueberry berries (4.45 mg/g d.m).

**Fig. 12:**
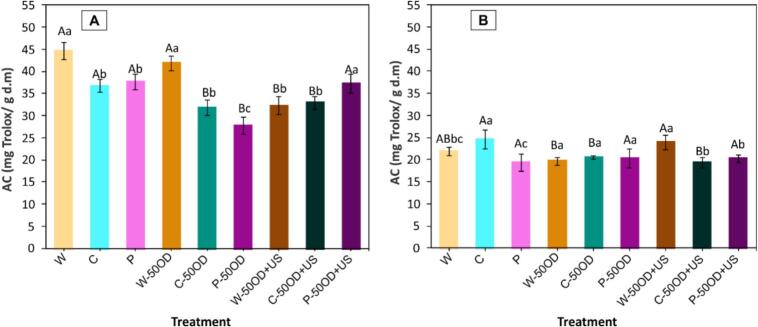
Antioxidant Capacity before (A) and after drying (B). Letters represent the Tukey’s mean comparison test (n = 6, α = 0.05): Upper-case letters compare among osmotic pre-treatment (without OD, 50OD and 50OD + US) and lower-case letters compare among W (whole), C (cut) and P (perforated).

Fig. 12 presents the antioxidant capacity (AC) of blueberries after different pre-treatments (Fig. 12.A) and after drying (Fig. 12.B). Regarding the effect of pre-treatments on the initial AC of whole blueberries, a similar trend was observed to that observed in TPC, where ultrasound-assisted OD pretreatment in mechanically modified samples allowed higher AC levels than when OD was applied without ultrasound. In these samples, the pre-treatment P-50OD + US best preserved antioxidant capacity, while the P-50OD treatment showed the lowest AC, with a decrease of 38%.

Compared to the initial AC of whole blueberries, the drying process led to a reduction in AC across all pre-treatments (Fig. 12.B), most notably in the P and C-50OD + US pre-treated samples, both exhibiting approximately 56% loss. Conversely, the treatments that best maintained AC after drying were C (45% degradation) and W-50OD + US (46.5% degradation). Furthermore, when comparing the AC of each pre-treatment after drying with its respective initial AC content (before drying), the P-50OD and W-50OD + US pre-treatments demonstrated the highest preservation rates, retaining over 73% of their initial AC levels. However, the greatest levels of degradation (>50%) were observed in whole samples without OD (W) and those subjected to OD (W-50OD). Although these samples did not undergo MM or structural changes due to ultrasound, they experienced prolonged drying times, which contributed substantially to the loss of antioxidant capacity (AC). This suggests that the variation in AC, as with total phenolic content (TPC) and total monomeric anthocyanins (TMA), depends on the degree of structural modifications and exposure to deteriorating conditions.

Summarizing, the observed decrease in TPC, TMA, and AC following pre-treatments was attributed to the exposure of bioactive compounds to deteriorative factors such as oxygen and light, particularly in the case of cutting and perforation treatments. Additionally, leaching bioactive compounds may have occurred during OD of mechanically modified samples (C and P). However, the application of ultrasound-assisted OD in these samples promoted higher levels due to a possible improvement in the extractability of bioactive compounds. Finally, reductions in bioactive compounds after drying have also been observed in dehydrated blueberries. However, in the present study, the levels of reduction were lower than those reported by Liu, Xie, Zielinska, Pan, Wang, Deng, Wang and Xiao [41], Zielinska and Michalska [57], and Zielinska and Michalska [58], who documented decreases of up to 76%, 77%, and 76% for total polyphenols, anthocyanins, and antioxidant capacity, respectively. Therefore, to obtain dried blueberries with an adequate content of TPC, TMA, AC, and that at the same time have reductions in drying time of between 19% − 29%, the pre-treatments P-50OD + US, and C-50OD were recommended.

### 3.6 Adsorption isotherm and shelf-life estimation

**Fig. 13:**
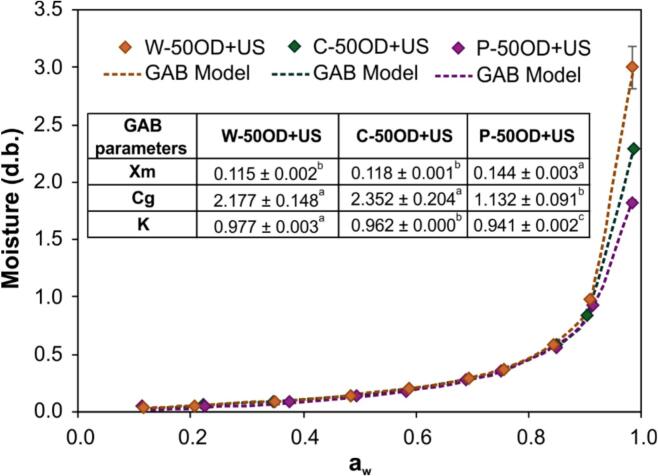
Water adsorption isotherm of dehydrated blueberry. The dots represent experimental data, while the dotted curves correspond to the data modeled using the GAB model. The merged table in the figure shows the parameters of the GAB model. Letters represent the Tukey’s mean comparison test (n = 2, α = 0.05).

Fig. 13 presents the water sorption isotherms of pre-treated and dried blueberries, along with the parameters of the GAB model used to describe them. The isotherm exhibits a Type III behavior, which is characteristic of products rich in sugars or polysaccharides, such as blueberries [59]. The monolayer moisture content (*X_m_*), calculated using the GAB model, represents the maximum amount of water tightly bound in the first layer of the material, which does not trigger spoilage reactions. The highest *X_m_* value was observed in the P-50OD + US treatment, indicating a greater capacity for retaining this first layer of water. The *C_g_* and *K* parameters are related to the energy of interaction between water in the monolayer and multilayer with the liquid water, respectively. The W-50OD + US and C-50OD + US treatments showed the highest *C_g_* values, reflecting a stronger interaction between water molecules and the surface sites. In all treatments, the *K* values were close to 1, suggesting that multilayer water is in a state like that of liquid water. Vega-Galvez, Lemus-Mondaca, Tello, Aranda and Yagnam [59] also note that *K* values near 1 are associated with high sugar solubility at aw ≥ 0.75. The GAB model parameters enabled the estimation of product shelf-life.

The shelf-life of dried blueberries was estimated (Equation (11) considering storage conditions of 25°C and 75% relative humidity, using three types of packaging. For the W-50OD + US dried blueberry, the estimated shelf-life was 1.32, 4.78, and 13.34 years for low-density polyethylene (LDPE), polymeric laminate (PL) (PE/adhesive/EVOH/adhesive/PE), and aluminum laminate (AL) packaging, respectively. For the C-50OD + US dried blueberry, the shelf-life was estimated at 0.9, 3.42, and 9.57 years for LDPE, PL, and AL packaging, respectively. For the P-50OD + US blueberry, the estimated shelf-life was 0.62, 2.26, and 6.32 months for LDPE, PL, and AL packaging, respectively. These results demonstrate that the products obtained with W-50OD + US and P-50OD + US treatments are the most stable regarding moisture increase, especially when aluminum laminate packaging is used. Indeed, aluminum laminate packaging has been shown to extend the shelf-life of dehydrated products for several years. For example, the shelf-life of dried peanuts stored at 25°C and 75% relative humidity exceeded 2.6 years when aluminum laminate packaging was used [37]. However, it is important to note that the shelf-life values obtained were estimations. Therefore, it is recommended to perform validations through real storage experiments.

Consequently, to extend the shelf-life of dried blueberries, it is recommended to use packaging materials such as PL and AL, which can maintain appropriate product moisture levels for several months or years. However, it should be considered that, during such prolonged storage, other undesirable reactions may occur, such as anthocyanin degradation or changes in sensory attributes, which should be considered in real shelf-life studies.

## 4 Conclusions

MM and OD, with or without ultrasound, were applied for improving drying kinetics and preserving blueberry quality. The Peleg model adequately described mass transfer during OD (R^2^ ≥ 0.838), showing that perforation increased equilibrium WL and SG, while cutting accelerated initial WL. Drying kinetics, fitted with the Page model (R^2^ ≥ 0.987), revealed that MM in combination with OD achieved drying time reductions between 16% and 32%. Viscoelastic measurements indicated that perforated berries lost skin elasticity drastically, while OD reduced both elasticity and viscosity due to the gain of solids and loss of water. Ultrasound-assisted OD, although not statistically significant, tended to further weaken the viscoelastic structure, especially in cut berries. Rehydration tests confirmed that mechanically modified berries absorbed water faster, whereas OD and ultrasound-assisted OD reduced the rehydration rate of berries because of a possible crust formation. Regarding bioactive compounds, in mechanically modified samples, the ultrasound-assisted OD allowed higher levels of total phenolics, anthocyanins, and antioxidant capacity after pre-treatment. Shelf-life estimation using the GAB model indicated that aluminum laminate packaging extended stability up to 13.34 years, while less effective packaging (low-density polyethylene) reduced stability to under 1.32 years. Overall, pre-treatments improved the convective drying time of blueberries while maintaining their post-drying properties at levels higher than those of whole blueberries (without pre-treatment). Specifically, perforation followed by US-assisted OD (50°Brix) (P-50OD + US) is the recommended pre-treatment, with potential application to valorize discarded blueberries. This approach maintains good rehydration properties, an adequate level of bioactive compounds and antioxidant capacity, while also reducing drying time.

Among the main limitations of this study, the experiments were conducted on a single blueberry cultivar (*Vaccinium corymbosum L.*) under laboratory-scale conditions, which may not fully represent industrial drying environments. Therefore, for future research, it is suggested to evaluate other blueberry cultivars since they may respond differently due to variations in skin morphology, wax composition, and phenolic profile, potentially affecting mass transfer and bioactive compound stability. In addition, conducting shelf-life studies of dried blueberries under real experimental storage conditions would be desirable, considering other factors, such as sensory attributes.

Regarding the pre-treatments explored in this study, mechanical modification — particularly perforation — proved effective in improving convective drying performance. Although perforation was implemented at laboratory scale using a prototype device, scaled-up systems based on rotating drums with pin arrays could enable continuous-flow industrial processing, representing a feasible engineering pathway worth exploring. Concerning ultrasound-assisted osmotic dehydration, some limitations should be considered in future studies and scale-up efforts. First, acoustic cavitation may negatively affect bioactive compounds through the generation of reactive oxygen species (ROS) and localized temperature increases, potentially accelerating oxidative degradation. Second, the excessive solid gain promoted by ultrasound during osmotic dehydration can be counterproductive in subsequent convective drying, as the resulting surface crust may hinder moisture evaporation and increase drying time, as observed for whole blueberries in this study (W-50OD + US). Finally, the ultrasound parameters used (33.86 W/L, 37 kHz) were optimized for a laboratory-scale bath; industrial implementation would require acoustic field modeling to ensure uniform cavitation energy distribution across larger processing volumes.

## CRediT authorship contribution statement

**Meliza Lindsay Rojas:** Writing – review & editing, Writing – original draft, Visualization, Supervision, Project administration, Methodology, Investigation, Funding acquisition, Formal analysis. **Maria Namoc:** Writing – review & editing, Writing – original draft, Methodology, Investigation, Data curation, Conceptualization. **Karla Ramirez:** Writing – review & editing, Writing – original draft, Methodology, Investigation, Data curation. **Alberto Claudio Miano:** Writing – review & editing, Writing – original draft, Visualization, Funding acquisition, Formal analysis, Conceptualization.

## 5 Funding

The financial support for this project provided by the “Consejo Nacional de Ciencia, Tecnología e Innovación Tecnológica” (CONCYTEC, Perú) through the “Programa Nacional de Investigación Científica y Estudios Avanzados” (ProCiencia, Perú) within the framework of the call “E041-2024–01”, according to contract N° PE501086399-2024-PROCIENCIA.

## Declaration of competing interest

The authors declare that they have no known competing financial interests or personal relationships that could have appeared to influence the work reported in this paper.
